# Incentive contract design considering quotas production: A principal-agent perspective

**DOI:** 10.1016/j.heliyon.2024.e24137

**Published:** 2024-01-09

**Authors:** Sen Liu, Lei Wang, Xuejiang Shi, Shibo Ouyang, Lifan Yang

**Affiliations:** a School of Logistics and Management Engineering, Yunnan University of Finance and Economics, Kunming, 650221, China; b Hongyun Honghe Tobacco (Group) Co., Ltd, Kunming, 652300, China; c Faculty of Management and Economics, Kunming University of Science and Technology, Kunming, 650093, China

**Keywords:** Bilateral efforts, Principal-agent decision theory, Information asymmetry, Production quotas

## Abstract

The quality of primary products in a supply chain is determined by the agent and the principal. Simultaneously, there are quota production constraints on the principals. Our discourse centers on the design of incentive contracts for agents within these supply chains. We derived the parameters describing the contract, level of effort, profits for both sides, and the minimum requirements of the principal for the proportion of high-quality primary products. This study compares the decision-making paradigms of agents and principals in various contexts. The results show that decision-making mechanisms are strongly influenced by individual effort costs, various internal and external organizational variables, and the interplay of efforts on both sides. Using numerical experiments, we investigate the effects of different situations between clients and contractors on contracting and effort strategies. The results show that when the agent exerts unilateral effort, reasonable incentive contracts can spur the agent to increase effort and thus increase the proportion of high-quality primary products. In the case of bilateral efforts, a well-calibrated contract design benefits the agent (bearing less risk). For the principal, the expected profit increases in most cases. When considering the quota production, it is necessary to set an appropriate limit on the proportion of high-quality primary products.

## 1 Introduction

Quota production, in which the government or an entity limits production or supply, is set below the producer's capacity. Initially, quota production systems were primarily used to achieve higher long-term profits [1] or protect limited resources [2].

Quota production systems are widely used in various industries. For example, milk producers adopted a quota production system in the agricultural product supply chain to obtain higher sustainable prices [3]. In some manufacturing industries, enterprises implement quota production systems for specific processes with higher production speeds to balance production among different processes [4]. In fisheries and mining, quotas are set to protect resources and prevent the exploitation and harvesting of limited or nonrenewable resources [2,[5], [6], [7]]. Low-carbon supply chains and greenhouse gas (GHG) emissions reductions have recently received widespread attention as environmental awareness grows [[8], [9], [10]]. Many countries have introduced point policies for manufacturers to reduce the use of fueled vehicles. Only manufacturers that produce and sell sufficient electric vehicles receive sufficient points to produce the corresponding number of fueled vehicles [11]. For instance, one of the businesses of the Geely Group (http://zgh.com/), based in Zhejiang, China, produces fuel-efficient family passenger cars. Given China's escalating regulations on emissions reduction and electric vehicle credit policies, Geely's conventional fuel vehicle production has faced stringent quotas.

Consequently, Geely Group collaborated with upstream component suppliers to jointly enhance component quality and reduce emissions. Similarly, under the auspices of the China National Grain Storage (CNGS) Group, grain silos have entered into partnerships with local agriculturists to augment the percentage of superior-grade wheat procured and stored. Carbon quotas, introduced as a mitigation strategy against greenhouse gas emissions, have found ubiquity across numerous nations and sectors [12,13].

Numerous global entities have shown interest in quota production, recognizing its potential to facilitate economic, environmental, and societal dividends. For example, quota production has been implemented in Canada to oversee the output of dairy, poultry, and other agricultural products [3,14,15]. In China, quota production is widely used in automobiles [16], mining [6], and other industries [17]. Moreover, under the latest environmental protection policies, carbon quotas are used in electricity, manufacturing, and other industries to reduce carbon emission targets [13,[18], [19], [20]]. OPEC has consistently upheld its crude oil production quota framework [21].

In production practice, quota production not only brings the aforementioned economic, environmental, and social dividends but also effectively promotes technological progress and improves product quality [22,23]. Several procurement entities adopted quotas for their cultivators within the agrarian supply chain. This strategy incentivizes agriculturists and agri-tech institutions to intensify their efforts to increase product quality.

The quality of the primary product (or raw material) is pivotal in the supply chain [24]. For instance, the cultivation of agricultural products depends on millions of small-scale farmers. However, the yield of the high-quality primary products is often low. For example, under traditional intensive cultivation, the yield of “first-grade” wheat, according to the standards of the China Grain Storage and Cereals Corporation, must have a capacity of 790 g/L, which is not high enough. Agricultural enterprises and research entities strive to elevate product quality via seed optimization, innovative cultivation techniques, and other interventions [25,26].

In the field of operations and supply chain management, many scholars have also paid attention to quota supply chains [27,28]. These chains diverge from their traditional counterparts in two ways. 1) On the demand side, the market price is determined by unified pricing (external managers rather than the product supplier) rather than the relationship between supply and demand. 2) On the supply side, the source of raw materials (agricultural products) is specialized planting and a single source. The principal or specialized agencies provide the seeds and technology for primary product planting; as a result, agricultural product planting or an improvement in agricultural product quality requires not only the efforts of the agent but also the costs of the principal [25]. Notably, this is a principal-agent relationship with bilateral efforts.

The first of the existing studies on the principal-agent relationship considering quotas considered cost limits [29], jointly defining the agents' costs. This differs from our focus on quota production. Other studies have prioritized production quotas [[30], [31], [32]]; our model integrates principals’ effortful actions, an element absent in their assessments. Existing studies have also touched on output or bonus quotas [33,34], but they diverge fundamentally from our model; they limit the maximum output, whereas our production quotas set the minimum.

Synthesizing practical observations with the extant literature, we posit that production quota supply chain incentives face inherent challenges. We divided these challenges into three pivotal questions.

(1) In the case of agents' unilateral efforts, can principals construct mechanisms that galvanize agents to enhance the proportion of high-quality primary products?
(2) Within a bilateral effort scenario involving the agent and principal, what is the incentive design for the principal? Do efforts in this supply chain eclipse those in a unilateral effort scenario?
(3) How do production quota frameworks modulate principal incentives? Does this paradigm imperil principals' anticipated profit margins?

The remainder of this paper is organized as follows. Section 2 provides an overview of related literature. Section 3 introduces the model and the notations used in the study. Section 4 analyzes the design of the incentive contract in three cases: unilateral effort by the agent, bilateral effort by the agent and principal, and production quota restrictions. Section 5 presents the numerical examples and analyses of the proposed model. Section 6 presents the managerial implications and conclusions.

## 2 Literature review

This study analyzes the role of incentives in supply chains with production quotas in producing high-quality products and generating profits. Next, we review the literature on production quotas and principal-agent theory and emphasize the contributions of our study to this literature.

### 2.1 Production quotas

Quota-production systems are widely used in many industries and interest researchers. Chen et al. [35] probed the impact of export quotas on the production and inventory policies of exporting companies and concluded that the costs associated with strictly adhering to current quotas can be substantial. Using balanced panel data on key minerals in China from 1999 to 2017, Yi et al. [6] discovered that the production quota policy encumbered profitability and bolstered the sustainable provision of critical minerals. Jensen and Vestergaard [36] scrutinized the implications of taxing fishing efforts amid information asymmetry. Aparicio et al. [37] evaluated the efficiency issues of regulated decision-making entities operating under quotas through data envelopment analysis and found that the quota system may have a significant impact on their performance. Cechura et al. [38] concluded that abolishing milk quotas had a positive effect. By analyzing quota policy and its operation in the British marine fishing industry, Cross [39] found low integration efficiency. Increasing attention to environmental issues has introduced carbon emission quotas into many industries and countries, such as production strategies that consider carbon quotas [18,20,40]. For instance, automotive sectors in several nations grappling with the ramifications of greenhouse gases have instantiated fuel–vehicle production quotas [11,16]. Wang et al. [31] integrate knowledge sharing into cross-border supply chains within a minimum constraint scenario and probe their inherent incentives. Li et al. [41] constructed a two-tiered principal-agent model that encapsulated governments, manufacturers, and suppliers to formulate contracts that fostered collaborative mitigation strategies.

Most of the aforementioned studies considered production decisions or performance analysis under the premise of quotas (e.g., internal production quotas, external carbon quotas, and tariff quotas), whereas our exploration pivots toward the design of incentives for firms in supply chains within the production quota bound.

Although quota production systems are widely used in many industries, few studies on supply chain incentives exist. Existing research mainly focuses on the impact of the quota system on production. In contrast, this study designs the incentive mechanism of the principal to the agent under the consideration of the quota system, which effectively improves the quality and output of the final products.

### 2.2 Principal-agent theory

Principal-agent theory has been widely used in modern enterprise production and government management. The principal-agent problem has garnered substantial attention in supply chain management. This mainly involves delegates working with other parties in the supply chain as downstream manufacturers [41]. Hidden information and adverse selection are two widely discussed principal-agent problems. For example, in the tourism supply chain, clients (i.e., cruise lines) rely too much on agents to create demand. The increasing number of cruise lines has led agents to overwhelm their clients [42]. Gong et al. [43] investigated the principal-agent model within parent-subsidiary dyads under information asymmetry and posited that optimal production modes and coordination can be achieved through judicious incentive contract design. For large-scale R&D projects, Liu and Ma [44] demarcated incentive models under supervision mechanisms with dual states of information symmetry and asymmetry, revealing that supervisory mechanisms can ameliorate information asymmetry in large-scale ventures. Yu et al. [45] found that within a principal-agent relationship with N agents, decentralized and centralized decision-making models remained inert in determining entities' effort levels and incentive coefficients. Li et al. [46] investigated the contract design problem of a virtual product supplier and a digital platform retailer when neither party knew the market demand. They ascertained that the optimal wholesale price, retail price, and level of sales effort investment depend on the retailer's level of risk aversion. Regarding principal and agent behavior, related studies on principal-agent theory can also be gleaned from reviews [47]. Zhang et al. [48] employed a principal-agent model to decode income distribution nuances within Chinese collective forest farms under the management of community forestry enterprises.

As mentioned above, the relevant research has widely considered the principal-agent problem. Moral hazards and adverse selection are the two most commonly discussed problems in the literature. In contrast to the above research, the quality of the intermediate product (or raw material) is also related to the efforts of both the agent and the principal (by providing better technical guidance, etc.). Furthermore, some studies have considered quotas in principal-agent models [20,36,41,48], focusing on the impact of an upper limit on the quantity produced, whereas this study focuses on the impact of a lower minimum supply limit. Finally, we consider the impact of implementing effortful actions, that is, bilateral efforts. This is widespread in some supply chains; for example, technical guidance from the principal in agricultural supply chains improves an agent's work.

## 3 Models

### 3.1 Model and assumptions

**Fig. 1:**
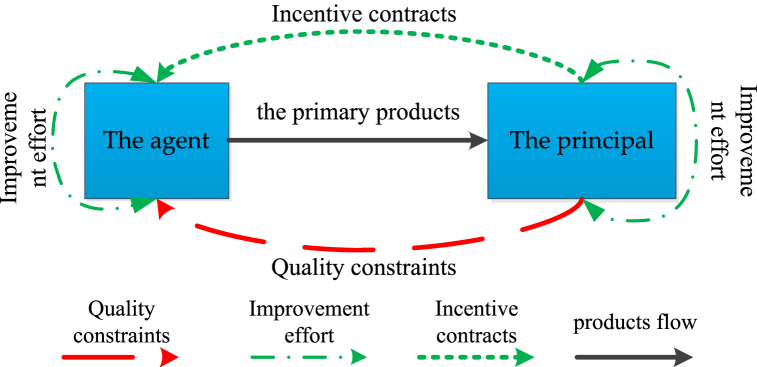
Principal-agent task relationships in supply chains with production quotas.

This study is based on a principal-agent model, as shown in Fig. 1, which consists of a buyer (principal) and a supplier (agent) [49]. An agent supplies primary products (or raw materials) to the principal. The principal processes primary products of different quality grades into final products of different quality grades and sells them in the terminal market. Higher-quality primary products lead to higher-quality products with higher brand value [50]. These high-quality products often result in higher profit margins for principals.

At the same time, considering that the principal faces a quota production situation, the total amount of end product produced by each principal may be limited, which often restricts the agent's high-quality primary production rate [4]. The quota production system does not restrict agents. However, the intermediate products it produces can only be sold to the principal; it must respond to (meet) the principal's quality requirements. The agent can improve the quality of intermediate products through enforcement efforts [51]; for example, farmers can improve the quality of their primary production through intensive farming. The principal is related to the quality of the agent's intermediate product [52]. For example, in the agricultural supply chain, in many commission production cooperation models, the buyer ensures the quality of the primary product by providing seeds and technology to the farmer.

Therefore, the agent and principal can improve product quality through efforts during planting and initial processing. In a typical principal-agent problem, the principal can observe the quality of the primary product but not the agent's effort [53]. In this context, the principal determines the optimal effort by designing a contract to stimulate the agent to maximize the principal's expected income. In turn, the agent chooses the appropriate effort level to ensure that the quality of the primary product meets the needs of the principal based on the contract parameters given by the factory.

The model is based on the assumptions below.

Assumption 1 The proportion of the agent's high-quality primary products to the total output is $\varphi \left(e_{F},e_{C}\right)$, where $\varphi \left(e_{F},e_{C}\right)=\varphi_{0}+k\left(e_{F}+e_{C}\right)$.

Assumption 2 The costs of implementing the efforts of the agent and principal are related to the degree of effort; please refer to the study of [53]; $c\left(e_{i}\right)=\frac{1}{2}\alpha_{i}{e_{i}}^{2},i=C,F$.

Assumption 3 The principal's purpose is to motivate the agent (including the principal in the case of bilateral efforts) to make greater efforts to improve the proportion of high-quality primary products at a reasonable cost. Similar to Ref. [31], this study assumes that incentive contracts are composed of fixed payments plus incentive payments, as demonstrated in Eq. (1):

(1) $$g\left(e_{F}\right)=A+\beta \varphi \left(e_{F},e_{C}\right)$$

### 3.2 Symbols and definitions

The symbols used throughout the study and their definitions are presented in Table 1.

**Table 1:** Model parameter settings.

Symbols	Definitions	Remarks
$\alpha_{i}$	The cost coefficient of the efforts of the agent or principal	i=F,C
$\varphi \left(e_{F},e_{C}\right)$	The percentage of high-quality primary products at the corresponding effort level	
k	The high-quality primary product and effortful action ratio factor	
$e^{\prime }$	The level of effort of the principal corresponding to the minimum limit of high-quality primary products	
$\bar{u}$	The unit income that the principal obtains from the high-quality end product	
$\underline{u}$	The unit income that the principal obtains from the low-quality end product	
$p_{1}$	The unit income that the agent obtains from the low-quality primary product	
$p_{2}$	The unit income that the agent obtains from the high-quality primary product	
$E\left(\pi_{F}\right)$	The agent's expected earnings	
$E\left(\pi_{C}\right)$	The expected benefits of the principal	
**Decision variables**	**Definitions**	**Remarks**
$e_{i}$	The degree of effort of the agent or principal	i=F,C
A	The fixed payment from the incentive contract given by the principal	
β	The incentive coefficient from the incentive contract given by the principal	

^1^ Note: In the following formula, the subscript F represents the agent and C represents the principal. The subscript fb indicates the unilateral efforts of the agent, sb indicates the bilateral situation of the agent and the principal, and tb indicates a situation with quality constraints.

## 4 Incentive contracts under scenarios of information asymmetry

In these scenarios, the principal cannot observe the agent's efforts. Therefore, the principal design contract menu encourages the agent to exert its best efforts to maximize the principal's expected benefits.

### 4.1 Situation of the unilateral efforts of the agent

In this scenario, the principal does not participate in quality improvement efforts; only the agent makes an effort to improve the quality of the primary product.

The expected benefits of the agent and principal after the incentive are:

(2) $$E\left(\pi_{F}^{fb}\right)=A^{fb}+\beta^{fb}\varphi \left(e_{F},0\right)+p_{1}q\varphi \left(e_{F},0\right)+p_{2}q\left(1-\varphi \left(e_{F},0\right)\right)-\frac{1}{2}\alpha_{F}{e_{F}}^{2}$$

(3) $$E\left(\pi_{C}^{fb}\right)=\bar{u}q\varphi \left(e_{F},0\right)+\underline{u}q\left(1-\varphi \left(e_{F},0\right)\right)-A^{fb}-\beta^{fb}\varphi \left(e_{F},0\right)$$

First, the agent must maximize profit by determining the optimal effort level based on the menu of contracts given by the principal:

(4) $$e_{F}^{fb}=\underset{e_{F}}{\mathrm{argmax}}\left\{\pi_{F}^{fb}\right\},\left(IC\right)$$

At the same time, under the principal's contract, it is necessary to meet the requirement that the agent's income be higher than its retained income. This study assumes that the retained benefits for the agent are the benefits received by the agent before it does not perform effortful actions; that is,

(5) $$\pi_{F}^{fb}\geq \pi_{F}^{0},\left(IR\right)$$

The principal problem is identifying the optimal menu of contracts that match these constraints.

(6) $$\begin{array}{l} \max_{\left(A,\beta \right)}\left\{E\left(\pi_{C}^{fb}\right)\right\}\\ s.t.\;\text{Constraints}\;\left(4-5\right) \end{array}$$

Proposition 1 *In the context of information asymmetry and the agent’s unilateral efforts*, *the optimal incentive system provided by the principal to the agent consists of*$A^{fb*}$and$\beta^{fb*}$. $A^{fb*}$ensures that the agent is willing to improve the process. *The*$\beta^{fb*}$ensures that both parties obtain their best benefits under these constraints. *Using this incentive mechanism*, $e_{F}^{*}$, $E\left(\pi_{F}^{fb*}\right)$, *and*$E\left(\pi_{C}^{fb*}\right)$*are expressed in* Eqs. (7), (8), (9): *All the proofs are provided in the appendix*.

(7) $$e_{F}^{*}=\frac{B}{\alpha_{F}}qk$$

(8) $$\left\{ \begin{array}{l} \beta^{fb*}=\left(\bar{u}-\underline{u}\right)q\\ A^{fb*}=\pi_{F}^{0}-Bq\varphi_{0}-\frac{1}{2}\frac{B^{2}}{\alpha_{F}}q^{2}k^{2}-p_{2}q \end{array}\right.$$

(9) $$\left\{ \begin{array}{l} E\left(\pi_{F}^{fb*}\right)=\pi_{F}^{0}\\ E\left(\pi_{C}^{fb*}\right)=Bq\varphi_{0}+\frac{1}{2}\frac{{\left(Bqk\right)}^{2}}{\alpha_{F}}+p_{2}q+\underline{u}q-\pi_{F}^{0} \end{array}\right.$$

Corollary 1 In a situation of information asymmetry and the unilateral efforts of the agent, $e_{C}$ is positively correlated with β.

Proof: Based on Eq. (24), we obtain $\frac{de_{F}^{fb*}}{d\beta }=\frac{k}{\alpha_{F}}\geq 0$. In other words, $e_{F}^{fb*}$ is an increasing function of the incentive coefficient.

Corollary 1 shows that, as β increases, the agent obtains more expected benefits and is more willing to perform effortful actions to improve the quality of the primary product. However, because the marginal benefits of the agent's hard work decrease, the principal will not infinitely increase β to motivate the agent to do its best. Under information asymmetry, the agent takes some risks and receives higher returns.

### 4.2 Bilateral efforts of the agent and the principal

Both parties simultaneously participate in quality improvement efforts in this scenario.

Under this circumstance, the expected profits of the agent and principal meet Eqs. (10), (11).

(10) $$E\left(\pi_{F}^{sb}\right)=A+\beta \varphi \left(e_{F},e_{C}\right)+p_{1}q\varphi \left(e_{F},e_{C}\right)+p_{2}q\left(1-\varphi \left(e_{F},e_{C}\right)\right)-\frac{1}{2}\alpha_{F}{e_{F}}^{2}$$

(11) $$E\left(\pi_{C}^{sb}\right)=\bar{u}q\varphi \left(e_{F},e_{C}\right)+\underline{u}q\left(1-\varphi \left(e_{F},e_{C}\right)\right)-A-\beta \varphi \left(e_{F},e_{C}\right)-\frac{1}{2}\alpha_{C}{e_{C}}^{2}$$

First, under the incentive constraint, the agent and the principal determine their respective e based on their expected return maximization conditions. That is,

(12) $$\left\{ \begin{array}{l} e_{F}^{sb}=\underset{e_{F}}{\mathrm{argmax}}\left\{\pi_{F}^{sb}\right\},\left(IC-1\right)\\ e_{C}^{sb}=\underset{e_{C}}{\mathrm{argmax}}\left\{\pi_{C}^{sb}\right\},\left(IC-2\right) \end{array}\right.$$

The requirement that $\pi_{F}$ be greater than retained earnings $\pi_{F}^{0}$ must also be satisfied under the contract provided by the principal; that is,

(13) $$\pi_{F}^{sb}\geq \pi_{F}^{0},\left(IR\right)$$

Finally, the principal problem is to find the optimal menu of contracts that satisfies the following constraints:

(14) $$\begin{array}{l} \max_{\left(A,\beta \right)}\left\{E\left(\pi_{C}^{sb}\right)\right\}\\ s.t.\;\text{Constraints}\;\left(12-13\right) \end{array}$$

Proposition 2 *In the cases of information asymmetry and bilateral efforts*, *when*$\alpha_{F}\leq 2\alpha_{C}$*is satisfied*, *the optimal incentive system provided by the principal to the agent comprises*$A^{sb*}$and$\beta^{sb*}$, *respectively*. $A^{sb*}$ensures that an agent is willing to improve the process. *The*$\beta^{sb*}$ensures that both parties obtain the best benefits under these constraints. *Under the incentive mechanism*, $e_{F}^{sb*}$, $E\left(\pi_{C}^{sb}\right)$, *and*$E\left(\pi_{F}^{sb}\right)$*are expressed in* Eqs. (15), (16), (17), *respectively*.

(15) $$\left\{ \begin{array}{l} e_{F}^{sb*}=\frac{\alpha_{C}B}{\left(\alpha_{F}+\alpha_{C}\right)\alpha_{F}}qk\\ e_{C}^{sb*}=\frac{\alpha_{F}B}{\left(\alpha_{F}+\alpha_{C}\right)\alpha_{C}}qk \end{array}\right.$$

(16) $$\left\{ \begin{array}{l} \beta^{sb*}=\frac{\alpha_{C}\left(\bar{u}-\underline{u}\right)-\alpha_{F}\left(p_{1}-p_{2}\right)}{\alpha_{F}+\alpha_{C}}q\\ A^{sb*}=\pi_{F}^{0}-\frac{\varphi_{0}Bq\alpha_{C}}{\left(\alpha_{F}+\alpha_{C}\right)}-\frac{B^{2}q^{2}k^{2}}{{\left(\alpha_{F}+\alpha_{C}\right)}^{2}}\frac{\alpha_{C}\left(2\alpha_{F}+\alpha_{C}\right)}{2\alpha_{F}}-p_{2}q \end{array}\right.$$

(17) $$\left\{ \begin{array}{l} E\left(\pi_{C}^{sb}\right)=\varphi_{0}q\frac{\alpha_{F}B}{\left(\alpha_{F}+\alpha_{C}\right)}+\frac{B^{2}q^{2}k^{2}}{{\left(\alpha_{F}+\alpha_{C}\right)}^{2}}\left(\alpha_{F}+\alpha_{C}-\frac{{\alpha_{F}}^{2}}{2\alpha_{C}}\right)+\underline{u}q-A^{sb*}\\ E\left(\pi_{F}^{sb}\right)=\varphi_{0}q\frac{\alpha_{C}B}{\left(\alpha_{F}+\alpha_{C}\right)}+\frac{B^{2}q^{2}k^{2}}{{\left(\alpha_{F}+\alpha_{C}\right)}^{2}}\frac{\alpha_{C}\left(2\alpha_{F}+\alpha_{C}\right)}{2\alpha_{F}}+p_{2}q+A^{sb*} \end{array}\right.$$

Corollary 2 Regarding information asymmetry and bilateral effort, the degree of an agent’s effort is positively correlated with β. $e_{C}$ is negatively correlated with β.

Proof: Based on Eq. (31), we obtain $\frac{de_{F}^{sb}}{d\beta }=\frac{k}{\alpha_{F}},\frac{de_{C}^{sb}}{d\beta }=\frac{-k}{\alpha_{C}}$; that is, the optimal effort of the agent is an increasing function of β, whereas $e_{C}$ is a decreasing function of β.

Corollary 2 shows that as β increases, the agent obtains more expected benefits and is more willing to perform actions to improve the primary product quality. The principal is unwilling to exert effort because more benefits are paid to the agent after the incentive. That is, an increase in β encourages the agent to engage in higher levels of hard work but also leads to the transfer of more benefits to the agent. The principal must find the right balance to maximize its benefits.

Corollary 3 *In a situation of information asymmetry and bilateral efforts*, $e_{F}$*and*$e_{C}$*are negatively correlated with the*α*of their own efforts and positively correlated with the*α*of the other party’s efforts*.

Proof: Based on Eq. (15), the first-order partial derivative of the effort cost coefficient can be obtained as $\left\{ \begin{array}{l} \frac{\partial e_{F}^{sb*}}{\partial \alpha_{F}}=-\frac{Bqk\alpha_{C}\left(2\alpha_{F}+\alpha_{C}\right)}{{\left(\alpha_{F}\alpha_{F}+\alpha_{C}\alpha_{F}\right)}^{2}}\leq 0\\ \frac{\partial e_{C}^{sb*}}{\partial \alpha_{F}}=\frac{Bqk\alpha_{C}\alpha_{C}}{{\left(\alpha_{F}\alpha_{C}+\alpha_{C}\alpha_{C}\right)}^{2}}\geq 0 \end{array}\right.$ $\left\{ \begin{array}{l} \frac{\partial e_{F}^{sb*}}{\partial \alpha_{C}}=\frac{Bqk\alpha_{F}\alpha_{F}}{{\left(\alpha_{F}\alpha_{F}+\alpha_{C}\alpha_{F}\right)}^{2}}\geq 0\\ \frac{\partial e_{C}^{sb*}}{\partial \alpha_{C}}=-\frac{Bqk\alpha_{F}\left(\alpha_{F}+2\alpha_{C}\right)}{{\left(\alpha_{F}\alpha_{C}+\alpha_{C}\alpha_{C}\right)}^{2}}\leq 0 \end{array}\right.$.

Corollary 3 is consistent with the intuitive interpretation that when the principal's or agent's α increases, this also implies an increase in effort cost, leading to a decrease in effort. When a party's α increases, its effort level decreases. If the other party wants to obtain a higher expected benefit, it must increase its effort to offset a decrease in the other party's effort.

Corollary 4 In the case of information asymmetry and bilateral efforts, compared to the unilateral effort scenario, the given β and $e_{F}$ are reduced. ΔA is increased. The proof is given in Eq. (18).

Proof:

(18) $$\left\{ \begin{array}{l} e_{F}^{sb*}-e_{F}^{fb*}=\frac{-Bqk}{\left(\alpha_{F}+\alpha_{C}\right)}\leq 0\\ \beta^{sb*}-\beta^{fb*}=-\frac{\alpha_{F}Bq}{\left(\alpha_{F}+\alpha_{C}\right)}\leq 0\\ A^{sb*}-A^{fb*}=\varphi_{0}Bq\frac{\alpha_{F}}{\left(\alpha_{F}+\alpha_{C}\right)}+\frac{B^{2}q^{2}k^{2}}{2\alpha_{F}}{\left(\frac{\alpha_{F}}{\alpha_{F}+\alpha_{C}}\right)}^{2}\geq 0 \end{array}\right.$$

Corollary 4 shows that, under the scenario of bilateral efforts, the incentive degree of the principal to the agent will be reduced. This is because an increase in β implies that the agent will take away more benefits. In particular, some of the benefits from the improvement in primary product quality created by the agent's efforts will also be removed, which will cause the principal to appropriately reduce incentives and improve profits. Because β is reduced for an agent, its efforts are reduced accordingly (see Corollaries 1 and 2). Correspondingly, as A increases and the incentive decreases, the risk borne by the agent decreases.

Corollary 5 *Under the scenario of information asymmetry and bilateral efforts*, *compared with the unilateral effort scenario*, *when*$\alpha_{F}\geq \alpha_{C}$*is met*, *the proportion of high-quality primary products increases*; *that is*, *the overall effort in the supply chain increases*, *and vice versa*.

Proof: $e_{F}^{sb*}+e_{C}^{sb*}-e_{F}^{fb*}=\frac{\alpha_{F}-\alpha_{C}}{\left(\alpha_{F}+\alpha_{C}\right)\alpha_{C}}Bqk$.

Corollary 5 shows that the overall level of effort in the supply chain may increase or decrease in the context of bilateral efforts. The total effort level gradually increases with an increase in $\alpha_{F}$. When it exceeds $\alpha_{C}$, the total effort of the supply chain exceeds that in the unilateral effort scenario. This is because agents are unwilling to exert a higher degree of effort to improve the quality of the primary product when the unit cost of effort is high. However, the principal is willing to implement a higher degree of effortful action because of the low unit cost of effort, which makes the overall effort of the supply chain higher than unilateral effortful actions. When $\alpha_{F}$ is lower than $\alpha_{C}$, the agent is willing to implement a higher e, but β will be lower (see Corollary 3). The benefits of the principal's efforts are transferred to the agent; therefore, the principal is unwilling to work hard.

This also shows that the agent and principal benefit from the efforts of both parties. The agent receives greater expected returns and reduces its risk (based on Eq. (13), the agent's expected returns do not increase, and the agent will only receive retained earnings) because the principal increases fixed payments and lowers incentive factors, which reduces the agent's risk.

### 4.3 The principal has restrictions on the proportion of high-quality primary products

In this context, the principal imposes a minimum limit on the proportion of high-quality primary products provided by the agent, that is, $\varphi \left(e_{F},e_{C}\right)\geq \underline{\varphi }$. Under this assumption, $\underline{\varphi }$ is transformed into $e^{\prime }$. Of course, because the principal determines $e_{C}$, the principal will not improve its efforts. Correspondingly, a pressure of effort was applied to the agent. Therefore, the agent's effort must be greater than or equal to $e^{\prime }$ (hereafter, collectively referred to as the effort constraint), which corresponds to the proportion of high-quality primary goods of the principal, as demonstrated in Eq. (19):

(19) $$e_{F}\geq e^{\prime }-e_{C}^{tb*}$$

where $e^{\prime }$ corresponds to the proportional constraints of high-quality primary products given by the principal.

When considering the constraints given by the principal, there are two situations.

1) When $e^{\prime }\leq e_{C}^{sb*}+e_{F}^{sb*}$, $e_{C}$ does not affect the model. The specific results are presented in Section 4.2.
2) When $e^{\prime }>e_{C}^{sb*}+e_{F}^{sb*}$, the model needs to add a level-of-effort constraint so that Eq. (14) can be rewritten as Eq. (20).

(20) $$\begin{array}{l} \max_{\left(A,\beta \right)}\left\{E\left(\pi_{C}^{tb}\right)\right\}\\ s.t.\left\{ \begin{array}{l} \pi_{F}^{tb}\geq \pi_{F}^{0},\left(IR-1\right)\\ e_{F}^{tb}\geq e^{\prime }-e_{C}^{tb*}\left(IR-2\right)\\ e_{C}^{tb}=\underset{e_{C}}{\mathrm{argmax}}\left\{\pi_{C}^{tb}\right\},\left(IC-1\right)\\ e_{F}^{tb}=\underset{e_{F}}{\mathrm{argmax}}\left\{\pi_{F}^{tb}\right\},\left(IC-2\right) \end{array}\right. \end{array}$$

Proposition 3 *When the principal’s efforts are met*, *that is*, $e^{\prime }>e_{C}^{tb*}+e_{F}^{tb*}$, *there is a unique optimal solution for*$e_{C}^{tb*}$and$e_{F}^{tb*}$, $\left(A^{tb*}，\beta^{tb*}\right)$, *and*$E\left(\pi_{F}^{tb}\right)$*and*$E\left(\pi_{C}^{tb}\right)$, *as shown in* Eqs. (21), (22), (23). *Appendix C presents the solution process*.

(21) $$\left\{ \begin{array}{l} \beta^{tb*}=\frac{\frac{e^{\prime }}{k}\alpha_{F}\alpha_{C}-\alpha_{C}q\left(p_{1}-p_{2}\right)-\alpha_{F}q\left(\bar{u}-\underline{u}\right)}{\left(\alpha_{C}-\alpha_{F}\right)}\\ A^{tb*}=\pi_{F}^{0}-\left(\frac{e^{\prime }\alpha_{C}-2\alpha_{F}B}{\left(\alpha_{C}-\alpha_{F}\right)}\right)\frac{\alpha_{F}\left(\varphi_{0}+ke^{\prime }\right)}{k}+\frac{1}{2}\alpha_{F}{\left(\frac{e^{\prime }\alpha_{C}-2\alpha_{F}B}{\left(\alpha_{C}-\alpha_{F}\right)}\right)}^{2}-p_{2}q \end{array}\right.$$

(22) $$\left\{ \begin{array}{l} e_{F}^{tb*}=\frac{e^{\prime }\alpha_{C}-kq\left(\bar{u}-\underline{u}+p_{1}-p_{2}\right)}{\left(\alpha_{C}-\alpha_{F}\right)}\\ e_{C}^{tb*}=\frac{kq\left(\bar{u}-\underline{u}+p_{1}-p_{2}\right)-e^{\prime }\alpha_{F}}{\left(\alpha_{C}-\alpha_{F}\right)} \end{array}\right.$$

(23) $$\left\{ \begin{array}{l} E\left(\pi_{F}^{tb}\right)=\left(\frac{e^{\prime }\alpha_{C}-2\alpha_{F}B}{\left(\alpha_{C}-\alpha_{F}\right)}\right)\frac{\alpha_{F}\left(\varphi_{0}+ke^{\prime }\right)}{k}-\frac{1}{2}\alpha_{F}{\left(\frac{e^{\prime }\alpha_{C}-2\alpha_{F}B}{\left(\alpha_{C}-\alpha_{F}\right)}\right)}^{2}+p_{2}q+A^{tb*}\\ E\left(\pi_{C}^{tb}\right)=\left(\frac{2\alpha_{F}B-e^{\prime }\alpha_{F}}{\left(\alpha_{C}-\alpha_{F}\right)}\right)\frac{\alpha_{C}\left(\varphi_{0}+ke^{\prime }\right)}{k}-\frac{1}{2}\alpha_{C}{\left(\frac{2\alpha_{F}B-e^{\prime }\alpha_{F}}{\left(\alpha_{C}-\alpha_{F}\right)}\right)}^{2}+\underline{u}q-A^{tb*} \end{array}\right.$$

From Proposition 3, we find that even if the agent is willing to continue to participate in the supply chain and $e_{C}^{tb*}$ is satisfied, $e^{\prime }>e_{C}^{tb*}+e_{F}^{tb*}$, the agent is willing to continue to participate in the supply chain. However, because the principal has an absolute advantage in the supply chain, it can also design a contract such that the agent obtains only retained earnings.

Considering the complexity of the function, we further analyze the impact of each endogenous and exogenous variable in the supply chain on both parties’ decisions and expected benefits through a numerical study in the next section.

## 5 Numerical study

To more intuitively explain the impact of various internal and external factors on incentive contracts in the presence of production quotas in the supply chain and compare the profits of each party under different models of information asymmetry, the bilateral efforts of agents and principals, and the proportion of demand for high-quality primary products, MATLAB was used to complete the numerical study and validate and analyze previous findings. This section presents the specific assignment of each parameter to the model. The numerical settings for the base case were based on the numerical analyses of Wang et al. [31], Sun et al. [54], and Zhang and Xu [55]. Drawing on these studies, we set the following parameters to focus on the response of our model: In the supply chain model, the initial values of the relevant parameters are $a_{C}=\left[1,6\right]$ be $a_{F}=9$, $\bar{u}=20$, $\underline{u}=10$, ρ=0.4, k=0.04, $\varphi_{0}=0.2$, $p_{1}=8$, $p_{2}=5$, $e^{\prime }=\left[7.5,8.5\right]$, and q=1000.

### 5.1 Impact of effort on the impact coefficient k of the high-quality primary product

**Fig. 2:**
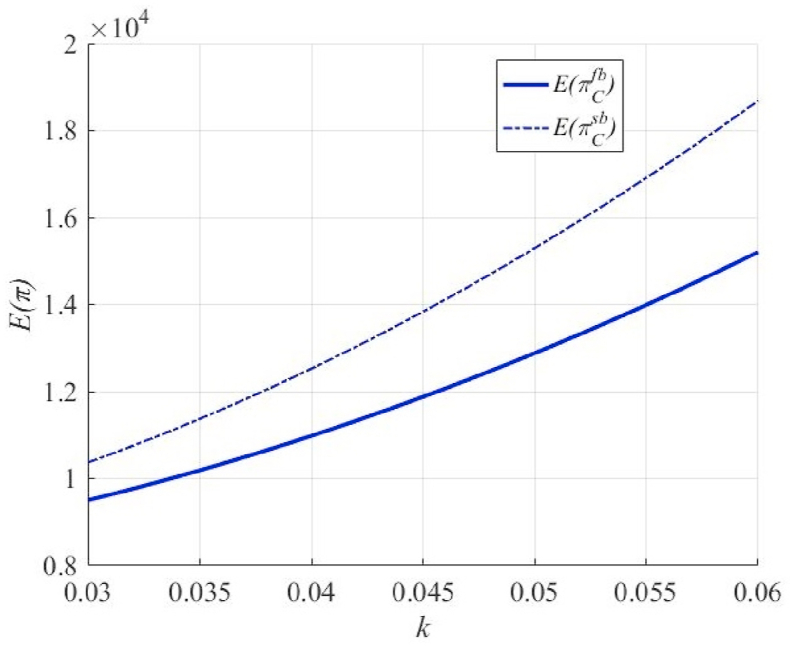
The effect of k on the principal's expected profit $E\left(\pi_{C}\right)$ in unilateral and bilateral effort scenarios.

As shown in Fig. 2, along with an increase in k, that is, a unit level of effort in exchange for a higher percentage of high-quality primary products, the expected return to the principal, as the principal, increases in both the unilateral and bilateral effort scenarios. Notably, the expected profits of the principal are higher in the bilateral effort case than in the unilateral effort case. This is mainly because the total effort in the supply chain is higher in the bilateral effort case, corresponding to a higher percentage of high-quality primary products. Simultaneously, with an increase in k, the expected profit of the principal increases faster in the case of a bilateral effort. In other words, the principal has a stronger incentive to exert an effort.

**Fig. 3:**
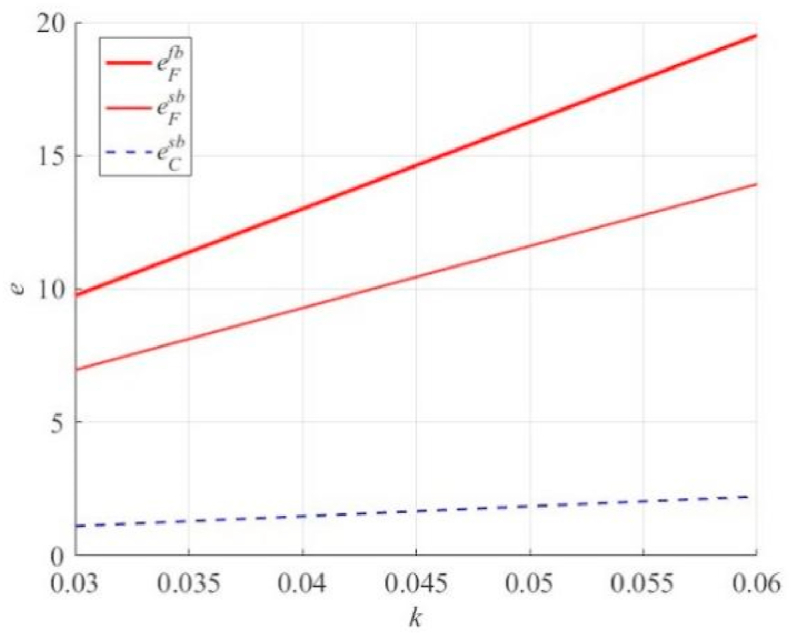
The effect of k on the principal's and agent's effort levels e under unilateral and bilateral effort scenarios (only the agent's effort level under unilateral effort).

Fig. 3 shows that as k increases, both the principal and the agent make higher-level efforts. However, contrary to our intuition, when $a_{F}<a_{C}$ occurs, the overall effort in the supply chain is smaller than in the case of bilateral efforts, and the efforts of the agent are lower (i.e., $e_{C}^{sb}+e_{F}^{sb}\leq e_{F}^{fb}$). In this case, the total degree of effort of the supply chain under the bilateral efforts of the principal and agent is lower than that under the unilateral effort scenario of the agent. In other words, the principal's execution effort reduces the total effort in the supply chain. The main reason is that the entrusting party and agent benefit the agent when they work hard. When the principal actively performs effortful actions, and the agent's effort-cost coefficient is high, the agent may adopt a free-riding strategy. A significant reduction in effort results in a relative reduction in the degree of total effort in the supply chain.

**Fig. 4:**
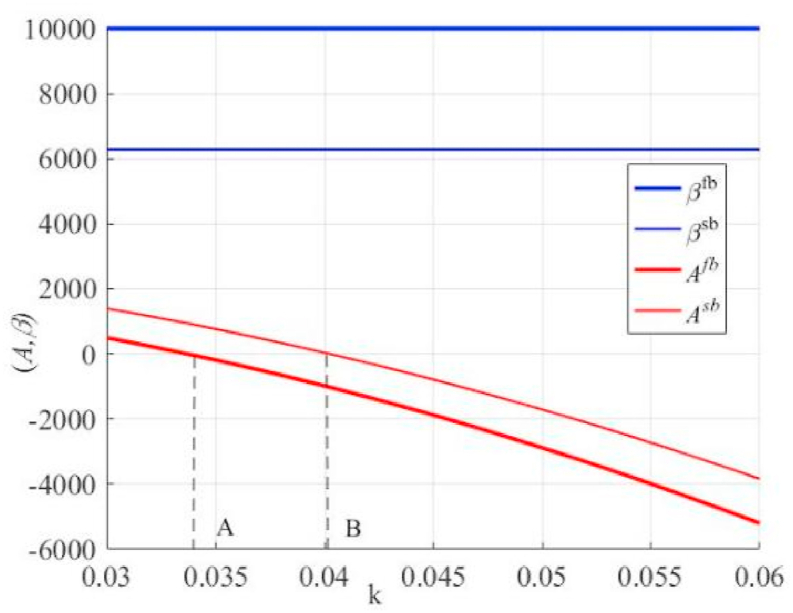
The effect of K on the fixed payment and incentive coefficient in contracts under unilateral and bilateral effort scenarios.

As shown in Fig. 4, the incentive coefficient in incentive contracts is not related to k, but fixed payments decrease as k increases. We also find that the principal is given a higher incentive coefficient in the unilateral effort case, corresponding to a lower fixed payment (see Corollary 3 for details of the relevant reasons). Furthermore, $A^{*}$ is positive when it is to the left of point A in the unilateral effort case or to the left of point B under bilateral payments; that is, the principal pays a fixed payment to the agent and then pays the incentive portion based on the percentage of the high-quality primary product that results from the agent's effort. By contrast, the agent pays a fixed payment to the principal, and the principal pays the incentive parts based on the proportion of high-quality primary products after the agent makes its efforts. This finding is in line with the reality. When the principal requires the agent to perform a process improvement effort and allows higher incentive remuneration, some agents will also be willing to pay part of the “guarantee” in advance. This is primarily determined by the agent's risk type. An agent with a risk preference may be more willing to accept such contracts, whereas risk avoidance types may be reluctant to accept such contracts.

### 5.2 Impact of the cost factor of effort in the cases of unilateral and bilateral efforts

**Fig. 5:**
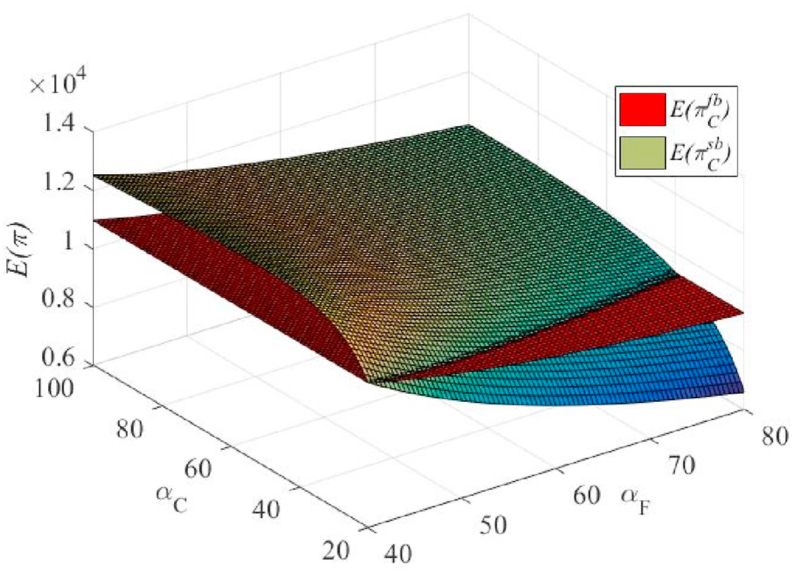
The effect of the cost coefficients of the agent's and principal's efforts on the principal's expected profit under the unilateral and bilateral effort scenarios.

From Fig. 5, we observe that under certain conditions, $E\left(\pi_{C}\right)$ is higher than that of unilateral efforts. This finding is in line with the intuitive understanding. However, under certain conditions, $E\left(\pi_{C}^{sb}\right)$ was lower than $E\left(\pi_{C}^{fb}\right)$. This finding is mainly due to the assumption that cooperation occurs in a scenario where the agent's cost factor is higher and the principal's cost factor is lower, $\alpha_{F}\geq \alpha_{C}$, which deliberately lowers the incentive factor to prevent the agent from sharing the benefits of its efforts. This could theoretically lead to a reduction in the total effort in the supply chain (see Fig. 7), such that $E\left(\pi_{C}\right)$ is reduced. In practice, this would not happen because the principal would not choose to perform effective actions at this point.

**Fig. 6:**
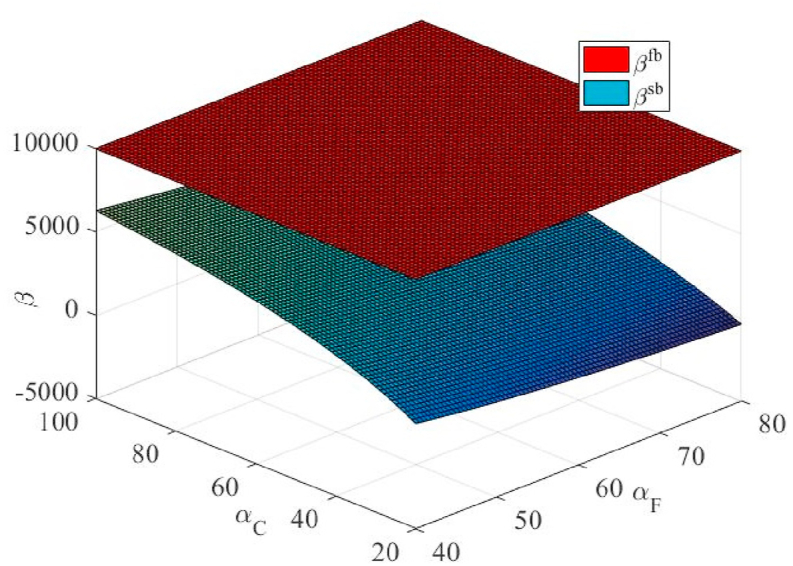
The effect of cost coefficients of agent and principal effort on contractual incentive coefficient in unilateral and bilateral effort scenarios.

**Fig. 7:**
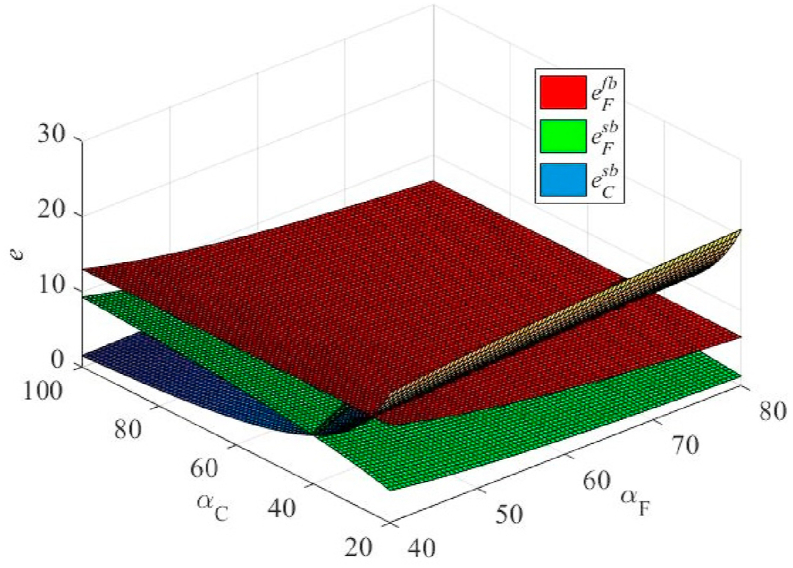
The effect of cost coefficients of agent and principal effort on effort degree under the unilateral and bilateral effort scenarios.

From Fig. 6, Fig. 7, we find that the principal gives a lower β than the unilateral efforts (which also explains why the profits in Fig. 5 are lower). In particular, as $e_{F}$ decreases, β also decreases. For specific reasons, please refer to the analysis presented in the previous section.

With an increase in $\alpha_{C}$, $e_{C}$ rapidly decreases. In contrast, the $e_{F}$ was slightly reduced. When $\alpha_{F}$ increases, $e_{C}$ also increases, whereas $e_{F}$ decreases slightly. This is because, when $e_{F}$ increases, the principal will naturally reduce its effort, whereas the agent must appropriately increase its effort to maximize its returns. Similarly, the agent obtained the corresponding result when $\alpha_{F}$ increased.

### 5.3 Changes in decision-making by all parties when the principal has minimum requirements for the proportion of high-quality primary products

**Fig. 8:**
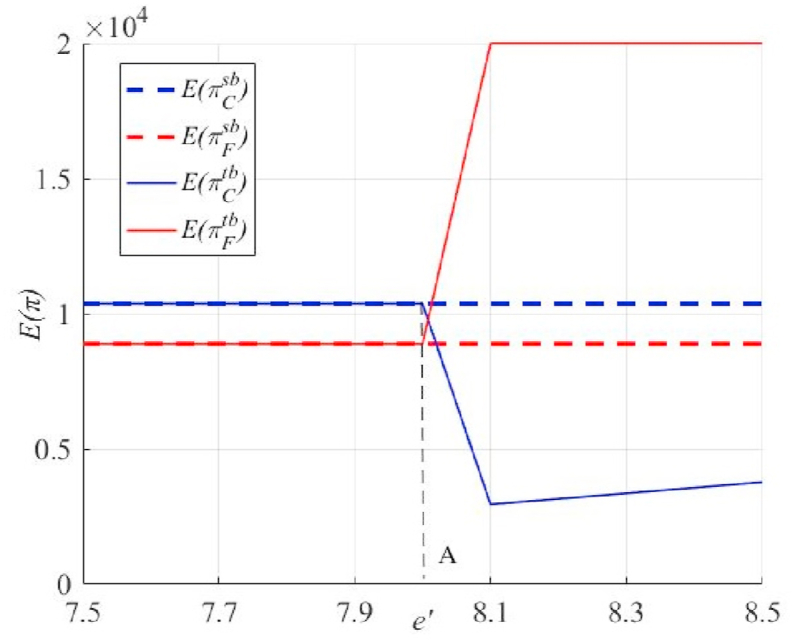
Impact of thresholds for the share of high-quality primary products of the principal under unilateral and bilateral endeavors on the expected profits of both parties.

**Fig. 9:**
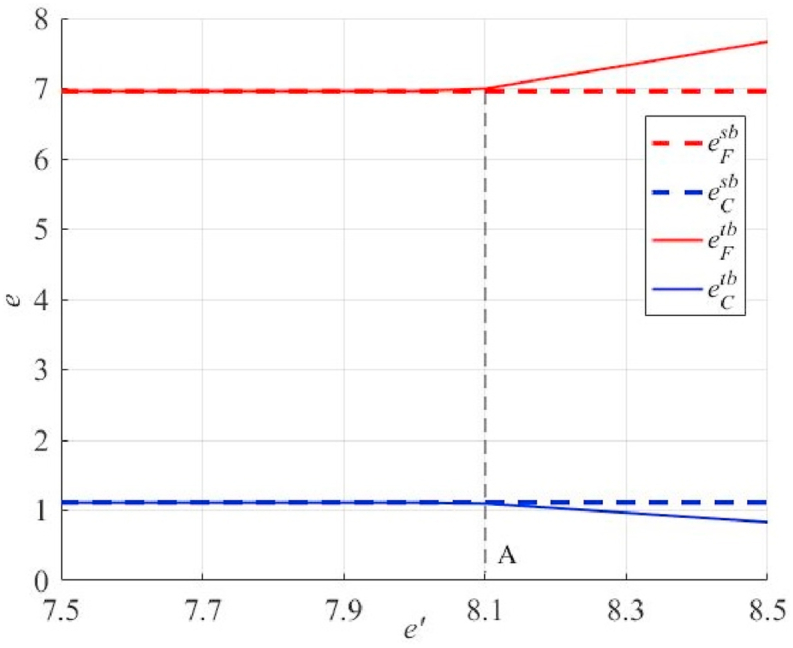
Impact of thresholds for the share of high-quality primary products of principals under unilateral and bilateral endeavors on the level of effort of the parties.

Based on Fig. 8, Fig. 9, it is clear that strict requirements for the principal's share of high-quality primary products will promote greater effort by the agent (see the right side of Point A in Fig. 9). However, this may not benefit principals in obtaining higher profits. This may even significantly lower the desired profit of the principal (see the right side of Point A in Fig. 8). The main reason is that both the principal and the agent have already made the optimal choice, and when the principal further increases k, β needs to be increased significantly to offset the loss of the agent (excess increase in effort costs). This also suggests that an excessively high k is not justified.

## 6 Conclusions

This study examines the design of incentive contracts for principals motivating agents in supply chains with information asymmetry and production quotas. Specifically, incentives aim to decide the optimal effort under conditions that maximize the principal's expected revenue. The models encompass the agent making a unilateral effort, the agent and the principal making a bilateral effort, and the principal having the minimum requirement for the share of high-quality primary products.

The key findings from this exploration, expanding upon the research questions tabled initially, include: 1) In unilateral effort scenarios, where the agent acts independently, the principal can stimulate the agent to make its best efforts through a reasonable menu design to maximize the principal's benefits. 2) Under the bilateral efforts of the agent and principal, the incentive coefficient given by the principal will be reduced, while the fixed payment will be increased, reducing the risk to the agent. However, the principal may not necessarily obtain greater expected benefits than the agent's unilateral efforts. In some cases, the principal expects higher returns when the agent makes unilateral efforts, whereas in other cases, the principal expects greater returns when the agent makes bilateral efforts. 3) Overly ambitious benchmarks the principal sets for high-quality primary products can backfire, sometimes resulting in net losses for the principal while ensuring that the agent remains profitable or at least insulated from losses. 4) In the absence of constraints on premium primary product proportions, a judicious incentive contract design can cultivate a win-win environment in which both parties prosper. When there is a constraint on the proportion of high-quality primary products, the agent's expected profit may increase significantly, and the corresponding risk increases significantly.

## 7 Several managerial implications can be drawn from the findings

(1) It is necessary to motivate the agent to exert process improvement efforts to increase the proportion of high-quality primary products. Incentive contracts can galvanize agents to increase their effort intensity, catalyzing an increase in the principal's desired revenue. (2) In a situation of information asymmetry, the principal making effortful actions may cause the agent to free-ride rather than increase its effort, which may reduce the principal's expected profits. That is, whether the principal needs to perform effortful actions should consider the possibility of free riding by the agent. (3) Even if a strict production quota is set by a regulator or a third party, the commissioner must determine a reasonable minimum percentage of quality primary products. If the percentage is unreasonable, it may be deleterious to interests. (4) Production quotas, although potentially detrimental to the principal's expected revenue, can be offset through judicious bilateral efforts. In other words, it is important for the agent to set an astute proportion of high-quality raw materials, choose a reasonable level of effort, and motivate the agent to execute the maximum effort echelon.

This study had several limitations that present opportunities for future research. First, the analysis was limited to single agents. However, involving multiple agents, such as those in an agricultural supply chain, is common. Therefore, future research should explore the design of incentive contracts in multiagent scenarios. In addition, it is important to consider the impact of production quotas on principals and agents. Our study examines only the case where production quotas exist on the principal side, neglecting the scenario in which agents face production quotas. Further exploration is warranted in future research.

## Funding

The work described in this paper was supported by the 10.13039/501100001809National Natural Science Foundation of China under grant Nos. 71862035 and 72372143; the Science and Technology project of Hongyun Honghe Tobacco (Group) Co., Ltd. under grant No. HYHH2023XX01; the Yunnan Provincial Academy and School Education Cooperation Project under grant No. SYSX202007; the Yunnan Fundamental Research Project under grant No. 202001AT070073; and the 21st Yunnan Young and Middle-aged Academic and Technical Leaders Reserve Personnel Training Program under grant No. 2019HB030.

## Data availability statement

The data used to support the findings of this study are available from the corresponding author upon request.

## CRediT authorship contribution statement

**Sen Liu:** Writing – original draft, Funding acquisition, Conceptualization. **Lei Wang:** Visualization, Investigation, Funding acquisition, Data curation. **Xuejiang Shi:** Validation, Software, Funding acquisition, Formal analysis, Data curation. **Shibo Ouyang:** Writing – original draft, Visualization, Resources. **Lifan Yang:** Writing – review & editing, Visualization, Software, Project administration, Methodology.

## Declaration of competing interest

The authors declare that there are no conflicts of interest regarding the publication of this paper.

## Appendix

Appendix A: Model solutions in the context of asymmetric information and unilateral efforts.

Based on Eq. (4) and its first-order conditions, we obtain

(24) $$e_{F}*=\frac{\beta +p_{1}q-p_{2}q}{\alpha }k$$

When the equal sign in Eq. (5) holds under the optimization conditions, by bringing Eqs. (24), (5) into Eq. (6), the objective function can be rewritten as

(25) $$E\left(\pi_{C}^{fb}\right)=-\frac{1}{2}\alpha_{F}{\left(\frac{\beta^{fb}+p_{1}q-p_{2}q}{\alpha_{F}}k\right)}^{2}-p_{1}q\varphi_{0}-p_{2}q\left(1-\varphi_{0}\right)+\underline{u}q+p_{2}q+\left(\bar{u}-\underline{u}+p_{1}-p_{2}\right)q\left(\varphi_{0}+k^{2}\frac{\beta^{fb}+p_{1}q-p_{2}q}{\alpha_{F}}\right)$$

The first and second derivatives of Eq. (25) are in accordance with Eq. (26):

(26) $$\left\{ \begin{array}{l} \frac{dE\left(\pi_{C}^{fb}\right)}{d\beta }=\left(\bar{u}-\underline{u}+p_{1}-p_{2}\right)q\frac{k^{2}}{\alpha_{F}}-\frac{\beta^{fb}+p_{1}q-p_{2}q}{\alpha_{F}}k^{2}\\ \frac{d^{2}E\left(\pi_{C}^{fb}\right)}{d\beta^{2}}=-\frac{1}{\alpha_{F}}k^{2}\leq 0 \end{array}\right.$$

Based on the first-order conditions,

(27) $$\beta^{fb*}=\left(\bar{u}-\underline{u}\right)q$$

By bringing Eq. (27) into Eq. (24), the agent's effort degree is

(28) $$e_{F}*=\frac{\bar{u}-\underline{u}+p_{1}-p_{2}}{\alpha_{F}}qk$$

Bringing Eqs. (27), (28) into Eqs. (2), (3) yields the expected benefits for the agent and the principal meet Eq. (29), respectively.

(29) $$\left\{ \begin{array}{l} E\left(\pi_{F}^{fb*}\right)=Bq\varphi_{0}+\frac{1}{2}\frac{B^{2}q^{2}k^{2}}{\alpha_{F}}+p_{2}q+A^{fb}\\ E\left(\pi_{C}^{fb*}\right)=Bq\varphi_{0}+\frac{1}{2}\alpha_{F}{\left(\frac{\bar{u}-\underline{u}+p_{1}-p_{2}}{\alpha_{F}}qk\right)}^{2}+p_{2}q+\underline{u}q-\pi_{F}^{0} \end{array}\right.$$

Further, it can be obtained that the fixed payment given by the principal party satisfies Eq. (30).

(30) $$A^{fb*}=\pi_{F}^{0}-\left(\bar{u}-\underline{u}+p_{1}-p_{2}\right)q\varphi_{0}-\frac{1}{2}\frac{{\left(\bar{u}-\underline{u}+p_{1}-p_{2}\right)}^{2}}{\alpha_{F}}q^{2}k^{2}-p_{2}q$$

## Appendix B Model solutions in the context of information asymmetry and bilateral efforts

Based on Eq. (12) and its first-order conditions,

(31) $$\left\{ \begin{array}{l} e_{F}^{sb}=\frac{\beta +p_{1}q-p_{2}q}{\alpha_{F}}k\\ e_{C}^{sb}=\frac{\bar{u}q-\underline{u}q-\beta }{\alpha_{C}}k \end{array}\right.$$

When the equal sign in Eq. (13) holds under the optimization condition, bringing Eqs. (31), (13) into Eq. (14), the objective function can be rewritten as

(32) $$E\left(\pi_{C}^{sb}\right)=\left(\bar{u}-\underline{u}+p_{1}-p_{2}\right)q\left(\varphi_{0}+k^{2}\left(\frac{\left(\alpha_{C}-\alpha_{F}\right)\beta +\left(p_{1}-p_{2}\right)\alpha_{C}q+\left(\bar{u}-\underline{u}\right)\alpha_{F}q}{\alpha_{F}\alpha_{C}}\right)\right)-\frac{1}{2}\frac{{\left(\bar{u}q-\underline{u}q-\beta \right)}^{2}k^{2}}{\alpha_{C}}-\frac{1}{2}\frac{{\left(\beta +p_{1}q-p_{2}q\right)}^{2}k^{2}}{\alpha_{F}}-\pi_{F}^{0}+p_{2}q+\underline{u}q$$

The first- and second-order derivatives of Eq. (32) are as shown in Eq. (33):

(33) $$\left\{ \begin{array}{l} \frac{dE\left(\pi_{C}^{sb}\right)}{d\beta }=\frac{\alpha_{C}\left(\bar{u}-\underline{u}\right)-\alpha_{F}\left(p_{1}-p_{2}\right)}{\alpha_{F}\alpha_{C}}qk^{2}-\frac{k^{2}}{\alpha_{C}}\beta -\frac{k^{2}}{\alpha_{F}}\beta \\ \frac{d^{2}E\left(\pi_{C}^{sb}\right)}{d\beta^{2}}=-k^{2}\left(\frac{1}{\alpha_{C}}+\frac{1}{\alpha_{F}}\right)\leq 0 \end{array}\right.$$

Based on the first-order condition, we obtain

(34) $$\beta *=\frac{\alpha_{C}\left(\bar{u}-\underline{u}\right)-\alpha_{F}\left(p_{1}-p_{2}\right)}{\alpha_{F}+\alpha_{C}}q$$

Taking Eq. (34) into Eq. (31), the efforts of agents and principals are as follows:

(35) $$\left\{ \begin{array}{l} e_{F}^{sb*}=\frac{\alpha_{C}\left(\bar{u}-\underline{u}+p_{1}-p_{2}\right)}{\left(\alpha_{F}+\alpha_{C}\right)\alpha_{F}}qk\\ e_{C}^{sb*}=\frac{\alpha_{F}\left(\bar{u}-\underline{u}+p_{1}-p_{2}\right)}{\left(\alpha_{F}+\alpha_{C}\right)\alpha_{C}}qk \end{array}\right.$$

The expected income of agents and principals can be obtained by bringing Eqs. (34), (35) into Eqs. (10), (11):

(36) $$\left\{ \begin{array}{l} E\left(\pi_{C}^{sb}\right)=\varphi_{0}q\frac{\alpha_{F}B}{\left(\alpha_{F}+\alpha_{C}\right)}+\frac{B^{2}q^{2}k^{2}}{{\left(\alpha_{F}+\alpha_{C}\right)}^{2}}\left(\alpha_{F}+\alpha_{C}-\frac{{\alpha_{F}}^{2}}{2\alpha_{C}}\right)+\underline{u}q-A\\ E\left(\pi_{F}^{sb}\right)=\varphi_{0}q\frac{\alpha_{C}B}{\left(\alpha_{F}+\alpha_{C}\right)}+\frac{B^{2}q^{2}k^{2}}{{\left(\alpha_{F}+\alpha_{C}\right)}^{2}}\frac{\alpha_{C}\left(2\alpha_{F}+\alpha_{C}\right)}{2\alpha_{F}}+p_{2}q+A \end{array}\right.$$

where $B=\left(\bar{u}-\underline{u}+p_{1}-p_{2}\right)$.

Eq. (36) is brought into Eq. (13), and the fixed payment satisfies Eq. (37).

(37) $$A^{sb*}=\pi_{F}^{0}-\frac{\varphi_{0}Bq\alpha_{C}}{\left(\alpha_{F}+\alpha_{C}\right)}-\frac{B^{2}q^{2}k^{2}}{{\left(\alpha_{F}+\alpha_{C}\right)}^{2}}\frac{\alpha_{C}\left(2\alpha_{F}+\alpha_{C}\right)}{2\alpha_{F}}-p_{2}q$$

## Appendix C Model solutions in the context of information asymmetry and given that the principal's efforts meet $e^{\prime }>e_{C}^{sb*}+e_{F}^{sb*}$

By bringing the principal's efforts in Eq. (31) into Eq. (20), the principal-agent model can be rewritten as

(38) $$\begin{array}{l} \max_{\left(A,\beta \right)}\left\{E\left(\pi_{C}^{tb}\right)\right\}\\ s.t.\left\{ \begin{array}{l} \pi_{F}^{tb}\geq \pi_{F}^{0},\left(IR-1\right)\\ e_{F}^{tb}\geq e^{\prime }-e_{C}^{sb*},\left(IR-2\right)\\ e_{C}^{tb}=\frac{\bar{u}q-\underline{u}q-\beta }{\alpha_{C}}k,\left(IC-2\right)\\ e_{C}^{tb}=\frac{\beta +p_{1}q-p_{2}q}{\alpha_{F}}k,\left(IC-2\right) \end{array}\right. \end{array}$$

According to Proposition 2, when the effort constraint of the principal is greater than the optimal effort in the supply chain, neither the principal nor the agent is willing to actively increase its effort, i.e., the effort in the supply chain is $e^{\prime }$. Based on restriction IC-1 from Eqs. (31), (38), we obtain

(39) $$e_{F}^{tb}+e_{C}^{tb}=\frac{\beta +p_{1}q-p_{2}q}{\alpha_{F}}k+\frac{\bar{u}q-\underline{u}q-\beta }{\alpha_{C}}k=e^{\prime }$$

Based on Eq. (39), we obtain

(40) $$\beta *=\frac{\frac{e^{\prime }}{k}\alpha_{F}\alpha_{C}-\alpha_{C}q\left(p_{1}-p_{2}\right)-\alpha_{F}q\left(\bar{u}-\underline{u}\right)}{\left(\alpha_{C}-\alpha_{F}\right)}$$

Bringing Eq. (40) into Eq. (31) yields the effort of the principal and the agent as follows:

(41) $$\left\{ \begin{array}{l} e_{F}^{tb*}=\frac{e^{\prime }\alpha_{C}-kq\left(\bar{u}-\underline{u}+p_{1}-p_{2}\right)}{\left(\alpha_{C}-\alpha_{F}\right)}\\ e_{C}^{tb*}=\frac{kq\left(\bar{u}-\underline{u}+p_{1}-p_{2}\right)-e^{\prime }\alpha_{F}}{\left(\alpha_{C}-\alpha_{F}\right)} \end{array}\right.$$

Bringing Eq. (41) into Eq. (38) yields the expected returns of the principal and the agent, respectively, as

(42) $$\left\{ \begin{array}{l} E\left(\pi_{F}^{tb}\right)=\left(\frac{e^{\prime }\alpha_{C}-2\alpha_{F}B}{\left(\alpha_{C}-\alpha_{F}\right)}\right)\frac{\alpha_{F}\left(\varphi_{0}+ke^{\prime }\right)}{k}-\frac{1}{2}\alpha_{F}{\left(\frac{e^{\prime }\alpha_{C}-2\alpha_{F}B}{\left(\alpha_{C}-\alpha_{F}\right)}\right)}^{2}+p_{2}q+A\\ E\left(\pi_{C}^{tb}\right)=\left(\frac{2\alpha_{F}B-e^{\prime }\alpha_{F}}{\left(\alpha_{C}-\alpha_{F}\right)}\right)\frac{\alpha_{C}\left(\varphi_{0}+ke^{\prime }\right)}{k}-\frac{1}{2}\alpha_{C}{\left(\frac{2\alpha_{F}B-e^{\prime }\alpha_{F}}{\left(\alpha_{C}-\alpha_{F}\right)}\right)}^{2}+\underline{u}q-A \end{array}\right.$$

By inserting Eq. (42) into the constraint IR from Eq. (20), the fixed payment is

(43) $$A^{tb*}=\pi_{F}^{0}-\left(\frac{e^{\prime }\alpha_{C}-2\alpha_{F}B}{\left(\alpha_{C}-\alpha_{F}\right)}\right)\frac{\alpha_{F}\left(\varphi_{0}+ke^{\prime }\right)}{k}+\frac{1}{2}\alpha_{F}{\left(\frac{e^{\prime }\alpha_{C}-2\alpha_{F}B}{\left(\alpha_{C}-\alpha_{F}\right)}\right)}^{2}-p_{2}q$$
